# Survival Outcomes in a Pediatric Antiretroviral Treatment Cohort in Southern Malawi

**DOI:** 10.1371/journal.pone.0165772

**Published:** 2016-11-03

**Authors:** Jason C. Brophy, Michael T. Hawkes, Edson Mwinjiwa, Gabriel Mateyu, Sumeet K. Sodhi, Adrienne K. Chan

**Affiliations:** 1 Dignitas International, Zomba, Malawi; 2 Department of Pediatrics, University of Ottawa, Children’s Hospital of Eastern Ontario, Ottawa, Canada; 3 Department of Pediatrics, University of Alberta, Stollery Children’s Hospital, Edmonton, Canada; 4 Zomba Central Hospital, Ministry of Health, Zomba, Malawi; 5 Department of Family and Community Medicine, University of Toronto, University Health Network, Toronto, Canada; 6 Department of Medicine, University of Toronto, Sunnybrook Hospital, Toronto, Canada; UCL Institute of Child Health, University College London, UNITED KINGDOM

## Abstract

**Background:**

Pediatric uptake and outcomes in antiretroviral treatment (ART) programmes have lagged behind adult programmes. We describe outcomes from a population-based pediatric ART cohort in rural southern Malawi.

**Methods:**

Data were analyzed on children who initiated ART from October/2003 –September/2011. Demographics and diagnoses were described and survival analyses conducted to assess the impact of age, presenting features at enrolment, and drug selection.

**Results:**

The cohort consisted of 2203 children <15 years of age. Age at entry was <1 year for 219 (10%), 1–1.9 years for 343 (16%), 2–4.9 years for 584 (27%), and 5–15 years for 1057 (48%) patients. Initial clinical diagnoses of tuberculosis and wasting were documented for 409 (19%) and 523 (24%) patients, respectively. Median follow-up time was 1.5 years (range 0–8 years), with 3900 patient-years of follow-up. Over the period of observation, 134 patients (6%) died, 1324 (60%) remained in the cohort, 345 (16%) transferred out, and 387 (18%) defaulted. Infants <1 year of age accounted for 19% of deaths, with a 2.7-fold adjusted mortality hazard ratio relative to 5–15 year olds; median time to death was also shorter for infants (60 days) than older children (108 days). Survival analysis demonstrated younger age at ART initiation, more advanced HIV stage, and presence of tuberculosis to each be associated with shorter survival time. Among children <5 years, severe wasting (weight-for-height z-score </ = -3.0) was also associated with reduced survival.

**Conclusions:**

Cumulative incidence of mortality was 5.2%, 7.1% and 7.7% after 1, 3, and 5 years, respectively, with disproportionate mortality in infants <1 year of age and those presenting with tuberculosis. These findings reinforce the urgent need for early diagnosis and treatment in this population, but also demonstrate that provision of pediatric care in a rural setting can yield outcomes comparable to more resourced urban settings of poor countries.

## Introduction

Pediatric HIV remains a major challenge to the health of children globally. Many advances in pediatric HIV treatment and programming have been realized in recent years–better antiretroviral (ARV) formulations for children [1], universal antiretroviral therapy (ART) for all children under 2 years [2], expansion of early infant diagnosis programs [3, 4], and improved regimens for prevention of vertical transmission (PVT) [5]. Despite these improvements, however, pediatric treatment coverage has lagged behind that achieved in adult treatment programs. In 2013, the WHO reported that the number of children on ART had increased from 566,000 in 2011 to 630,000 in 2012, however the percentage increase was much lower than that for adults (10% versus 20%) [6]. Likewise, treatment provision to those eligible for ART was much less for children than for adults (34% versus 68%), as was the relative improvement from 2011 to 2012 (12% increase in pediatric coverage versus 22% increase in adults). These difficulties reflect the complexity of pediatric diagnosis and treatment, as well as the lack of pediatric HIV expertise and capacity in low-income country settings.

Malawi is a country in southern Africa that has made substantial progress in the battle against HIV, reducing its HIV prevalence from 14.6% a decade ago to a current estimate of 10.0% [7]. Malawi is presently estimated to have 930,000 people living with HIV/AIDS, including 130,000 children <15 years of age. Providing HIV care in general, and pediatric HIV care specifically, has been a struggle in a country with inadequate human resources for health [8, 9]. The Canadian medical and research NGO *Dignitas International* has been present in Zomba District in southern Malawi since 2004, working collaboratively with the Ministry of Health (MOH) to expand capacity for ART program delivery. In an attempt to ensure sustainable strengthening of local health systems, ART provision has adhered to the successive iterations of Malawi’s national HIV treatment guidelines. ART was initially provided exclusively at Zomba Central Hospital, a tertiary level referral centre in Zomba municipality (population 87,366) [10], but over time ART delivery has been successfully decentralized to 31 primary health centres within Zomba District (670,533) [10]. In total, over 35,000 patients are presently on ART with 80% receiving it at the primary care level. Pediatric patients constitute 10% of the overall cohort. Decentralization of pediatric ART in Zomba District has lagged behind that of adults, with a higher proportion of pediatric patients receiving care at ZCH (11% of the ZCH cohort) than at decentralized clinics (8% of the decentralized cohort) [11]. While the argument can be made that pediatric care often requires more specialized support that cannot be obtained at the primary health level, a simplified public health approach adhering to local provision of care favours eventual decentralization of such patients.

The current study was undertaken to describe care and treatment outcomes of pediatric patients in the Zomba District ART program. In addition, determinants of mortality and other clinical outcomes were characterized for children receiving care in this government-lead ART program, in order to better inform approaches to pediatric ART provision locally and in other HIV-endemic countries.

## Methods

A retrospective cohort analysis was undertaken. Subjects were HIV-infected children aged less than 15 years at the time of ART initiation residing in Zomba District and accessing care at one of the district’s 31 health facilities during the period of October 2003 to September 2011. Data on patient demographics, diagnoses, and outcomes were extracted from the *Zomba District Observational Database & Cohort Study* (Z-OCS), a surveillance project conducted by Dignitas International and funded by the Canadian Institutes of Health Research (CIHR) and United States Agency for International Development (USAID). Methods for data collection in this program have been previously described in detail [12]. In brief, Z-OCS collected information from post-visit review of standard Malawi Ministry of Health (MOH) ART monitoring tools. HIV care was implemented as per national MOH ART guidelines (according to sequential editions from 2003, 2006, and 2008), with ART indications based on WHO clinical staging or CD4 count where measurement of CD4 was available. National pediatric HIV diagnosis and ART recommendations were aligned with sequential WHO recommendations. Implementation of early infant diagnosis for children <18 months with DNA PCR and universal treatment for infants <12 months began in 2008 [13], although WHO recommendations for empiric diagnosis and treatment of infants based on serologic testing and advanced clinical status remained an option. The first line ART regimen in Malawi for the majority of the enrolment period was stavudine (d4T), lamivudine (3TC) and nevirapine (NVP), with pediatric liquid formulations or split adult pills in the beginning of the study period and then in pediatric fixed-dosed combinations as formulations became widely available. After initiation, follow-up was monthly and after approximately 6 months, patients could be followed less often depending on assessment of adherence and distance to travel for appointments. Decentralization of ART delivery for some patients began in 2007, at the discretion of the provider and receiving primary health centre; the traditional authority (which in Malawi is the term for a sub-district administrative unit or home community) for each patient was recorded, but whether and when decentralization occurred and to which specific primary health centre care was transferred were not captured reliably by MOH monitoring tools. Mortality outcomes were documented by clinic health care provider staff and by reports from trained patient guardians where deaths occurred at home; additional deaths were identified through routine defaulter tracing performed as per standard of care by Malawi MOH staff. Patients outcomes were classified as either: alive and in care; dead; defaulted; or transferred out. Defaulted was defined as per Malawi MOH ART Guidelines as having failed to appear within 2 months after the next appointment date [13].

Statistical analysis was performed using SPSS version 16.0 (SPSS, Chicago, IL). Demographic, clinical features and diagnoses at the time of ART initiation were described for the cohort using frequencies for categorical variables, and medians with interquartile ranges for continuous variables (non-Gaussian distribution). Height, weight and age were used to compute the weight-for-age, height-for-age, and weight-for-height z-scores, based on WHO normative data and syntax for SPSS for children < 5 years of age, for whom this indices are validated prognostic indicators [14]. Wasting, severe stunting, and very low weight-for-age were defined as weight-for-height, height-for age, and weight-for-age z-scores of -3.0 or less, respectively. WHO definitions of severe immunosuppression according to CD4 cell count thresholds for age were used, as per Malawi MOH ART Guidelines [13]. Mortality incidence rates were computed for the total observation period and for the first 12 months after initiation of ART, with Poisson confidence interval estimates. Survival analysis was performed using Kaplan-Meier plots, and using log-rank testing for differences between factor levels. Cases were right censored at the time of last encounter if they were lost to follow-up, transferred out of the cohort, or still being followed in the ART program.

Multivariable Cox regression models were explored to adjust for possible confounding between multiple co-variates using R (R Core Team, version 3.0.1, 2013) and *survival* package [15, 16]. These models were used to estimate hazard ratios (HRs) with 95% confidence intervals (CIs) to quantify the association between survival and baseline demographic and clinical characteristics/diagnoses. The primary model included all enrolled children (n = 2203). Time to death was the outcome variable. Censoring was performed as above. Independent variables were included in the model based on the univariate analysis if the p-value was less than 0.1. Variables were selected for retention in the model by backward selection. Once the primary model was chosen, a sensitivity analysis was undertaken to examine the effect of traditional authority (TA) as a proxy for decentralization site, since management and mortality may differ at different sites. The effect of two-way interaction terms was explored in the multivariable models. Subgroup analyses were performed to assess the robustness of model estimates in selected sub-populations. Competing risk modeling was performed using the R package *cmprsk* [17] and the function *CumIncidence* [18] to model the competing risks of death, transfer out of cohort, and defaulting.

Ethics approval was obtained for the Z-OCS study from the National Health Sciences Research Committee of Malawi and the University Health Network Research Ethics Board, University of Toronto, Canada.

## Results

A total of 2203 children below the age of 15 years were enrolled between October 2003 and Sept 2011. The median (range) age at enrolment was 4.8 (0–15) years and 1114 (51%) were female. The majority of children 1247 (68%) were WHO clinical stage 3 or 4 at initiation. Additional characteristics of the cohort are shown in Table 1.

**Table 1:** Baseline characteristics of Z-OCS pediatric cohort.

Patient Characteristic	Statistic (N = 2203)	
Age at entry, median (IQR)	4.8 (1.9–9.3)	
Age categories, n (%)	< 1 year	219 (10)
	1 - <2 years	343 (16)
	2 - <5 years	584 (27)
	5–15 years	1057 (48)
Sex, n (% female)	1114 (51)	
Calendar year at enrolment in cohort, n (%)	2003–2005	76 (3.4)
	2006–2009	1308 (59)
	2010–2011	819 (37)
Traditional Authority, n (%)	Chikowi	751 (34)
	Mwambo	443 (20)
	Mlumbe	377 (17)
	Malemia	244 (11)
	Kumtumanji	188 (8.5)
	Zomba Town	23 (1.0)
	Other^1^	108 (4.9)
	unknown	69 (3.1)
Baseline clinical staging, n (%) (N = 1821)^2^	Stage 1	340 (19)
	Stage 2	324 (13)
	Stage 3	861 (47)
	Stage 4	386 (21)
Baseline absolute CD4 count, median (IQR) cells/uL (N = 223)^3^	< 1 year	260 (244–328)
	1 - <2 years	527 (250–774)
	2 - <5 years	357 (250–541)
	5–15 years	243 (137–343)
Diagnoses at enrollment, n (%)	Tuberculosis	409 (19)
	Oral candidiasis	241 (11)
Nutritional status at ART initiation, n (%) (N = 1112)^4^	Wasting^4^^,^^5^	109 (9.8)
	Stunting^4^^,^^6^	370 (33)
	Weight-for-age<-3SD^4^	354 (32)

^1^ The remaining sites contributed fewer than 10 patients each to the cohort.

^2^ Clinical staging was available for 1821 (83%) of cohort

^3^ 223 (10%) patients had CD4 count performed at enrolment

^4^ subgroup includes only children <5 years old, for whom indices of malnutrition are well validated

^5^ weight-for-length/height<-3SD

^6^ length/height-for-age<-3SD

WHO clinical stage was available for 1821 (83%) of the cohort at enrolment. Missing clinical stage data was more frequent in infants (23% of infants <1 yr *vs* 16% of children and youth 1 to 15 yrs, p = 0.012). There were small but statistically significant differences in missing clinical stage data between TAs, ranging from 13% for those from Zomba town to 21% for those from Mwambo (p = 0.013). Enrolment in earlier calendar years appeared to be associated with a higher proportion of missing clinical stage data (43% in 2003–5 *vs* 16% in 2006–11), although no significant trend with increasing calendar year was observed beyond 2006 (p = 0.40 for trend).

Baseline CD4 enumeration was performed on 223 (10%) subjects and severe immunosuppression was evident in 130 (58%). CD4 counts were performed less frequently on younger children (p = 0.003), on children with tuberculosis (3.9% versus 11%, p<0.001), and on those with oral candidiasis (5.0% versus 11%, p = 0.008). CD4 counts were obtained less frequently at baseline in fatal cases than in survivors (2/134 [1.5%] versus 155/1324 [12%], p<0.001). There were too few deaths (n = 2) among children who underwent CD4 testing to assess the relationship between CD4 count and mortality in this cohort.

In 1608 (73%) cases, treatment consisted of d4T/3TC/NVP at age-appropriate doses. Other common regimens included: zidovudine (AZT)/3TC/NVP [n = 289 (13%)]; d4T/3TC/efavirenz (EFV) [n = 55 (2.5%)]; or AZT/3TC/EFV [n = 8 (0.36%)]. Alternate regimens were used in fewer than 1% of cases.

The median (range) duration of follow-up was 1.5 (0–8) years, with a cumulative total of 3900 patient-years of follow-up. Over the period of observation, 134 (6.1%) patients died, 1324 (60%) remained actively followed in the cohort, 345 (16%) transferred out of the program and were alive at the last point of contact, and 387 (18%) defaulted; outcome data was missing for 13 patients. The overall mortality rate was 3.4 deaths per 100 patient years (PYs) (95%CI 2.9–4.0). Of the 134 deaths, 105 (78%) occurred in the first year after initiation of ART, with a 12-month mortality rate of 6.6 deaths per 100 PYs (95%CI 5.5–7.9). The median time to death was shorter for infants <1 year at 60 days compared to 108 days for older children.

**Fig 1:**
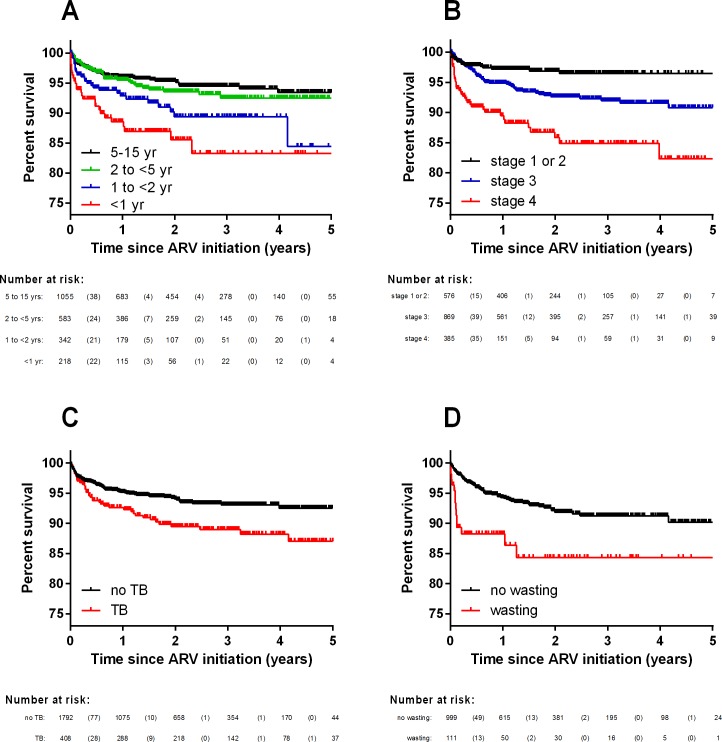
**Survival curves for the Z-OCS pediatric cohort (n = 2203) by (A) age at enrolment; (B) clinical stage at diagnosis; (C) clinical diagnosis of tuberculosis at enrolment; and (D) wasting (weight for-length/height <-3SD).** Survival analysis for wasting was restricted to patients <5 years of age.

Survival analysis (Kaplan-Meier) showed that younger age at ART initiation (Fig 1A, p<0.001), more advanced HIV stage at presentation (Fig 1B, p<0.001), and a diagnosis of tuberculosis (Fig 1C, p = 0.001) were associated with a shorter survival time. Documentation of oral candidiasis at the time of ART initiation was not associated with subsequent mortality (p = 0.26). Among children under 5 years, severe wasting (Fig 1D) and very low weight-for-age were both associated with a reduced survival time (p<0.001), although severe stunting was not (p = 0.36). There was no difference in survival according to year of inception into the cohort. In a univariate analysis, EFV-based ART regimens were associated with prolonged survival (p = 0.036); however, EFV was not prescribed to children under the age of 3 years and mortality was age-dependent. When the analysis was restricted to children aged 3 years and older, there was no statistically significant difference in survival according to EFV treatment (p = 0.11).

Results of the multivariable Cox proportional hazard analysis of the time to death are presented in Table 2. Independent variables in the model were age (categorical, 4 factor levels), WHO stage (categorical, 4 factor levels), and TB (binary). Visual inspection of log(-log(Survival)) curves did not reveal any obvious deviations from parallel between curves corresponding to each factor level. Plots of scaled Schoenfeld residuals did not reveal any obvious trends over survival time. Formal testing of the final model residuals using a global chi-square test [as described by Therneau and Grambsch [16] did not show evidence of deviation from proportional hazard assumption (chi-square = 9.3, p = 0.23).

**Table 2:** Predictors of survival in Z-OCS pediatric cohort.

Baseline characteristics	Total (N = 2203)	Died (n = 134)	Unadjusted Mortality HR (95% CI)	p-value	Adjusted Mortality HR (95% CI)^1^	p-value
Gender						
Female	1114	64 (5.7%)	1.0 (Reference)			
Male	1089	70 (6.4%)	1.1 (0.81–1.6)	0.46		
Age at ART initiation						
<1 year	219	26 (12%)	3.0 (1.9–4.9)	<0.001	2.7 (1.6–4.8)	<0.001
1-<2 years	343	27 (7.9%)	1.9 (1.2–3.1)	0.006	1.8 (1.1–3.1)	0.023
2-<5 years	584	33 (5.7%)	1.2 (0.80–1.9)	0.34	1.4 (0.86–2.2)	0.18
5–15 years	1057	48 (4.5%)	1.0 (Reference)		1.0 (Reference)	
Calendar year at ART initiation			0.93 (0.83–1.04)^2^	0.18		
2003–5	76	5 (6.6%)				
2006–9	1308	95 (7.3%)				
2010–11	819	34 (4.2)				
Traditional Authority						
Chikowi	751	48 (6.4)	1.0 (Reference)			
Mwambo	443	30 (6.8)	1.1 (0.72–1.8)	0.57		
Mlumbe	377	22 (5.8)	0.96 (0.58–1.6)	0.86		
Malemia	244	12 (5.0)	0.83 (0.44–1.6)	0.56		
Kumtumanji	188	14 (7.5)	1.3 (0.70–2.3)	0.45		
Zomba Town	23	3 (13)	1.8 (0.56–5.8)	0.32		
Other^3^	108	3 (2.9)	0.63 (0.19–2.0)	0.43		
unknown	69	2 (2.9)	0.59 (0.14–2.4)	0.47		
WHO stage at ART initiation						
I	342	12 (3.5%)	1.0 (Reference)		1.0 (Reference)	
II	235	5 (2.1%)	0.61 (0.21–1.7)	0.35	0.78 (0.27–2.2)	0.64
III	870	55 (6.3%)	1.8 (0.95–3.3)	0.073	1.5 (0.79–3.0)	0.21
IV	386	42 (11%)	3.9 (2.1–7.4)	<0.001	4.0 (2.1–7.7)	<0.001
Missing	370	20 (5.4%)				
Nutrition status at ART initiation						
Wasting^4^	109	15 (14%)	3.2 (1.7–5.9)	<0.001		
Stunting^5^	370	25 (6.8%)	1.3 (0.74–2.3)	0.36		
Weight-for-age<-3SD	354	37 (10%)	2.3 (1.4–3.6)	<0.001		
Co-infections at ART initiation						
TB	409	40 (9.8%)	1.7 (1.1–2.4)	0.007	2.2 (1.4–3.4)	0.001
Oral candidiasis	241	12 (5.0%)	0.71 (0.39–1.3)	0.26		

^1^ Cox proportional hazard model included age, HIV stage and tuberculosis at the time of ARV initiation as predictive variables of survival

^2^ Year of enrolment included as continuous variable in Cox proportional-hazard model.

^3^ The remaining sites contributed fewer than 10 patients each to the cohort.

^4^ weight-for-length/height<-3SD

^5^ length/height-for-age<-3SD

Several alternative multivariable models were explored to assess the robustness of model estimates in a sensitivity analysis (S1 Table). The primary model (Model 1, S1 Table), described above, identified age, stage and TB co-infection as key predictors of mortality in the cohort. We included TA as a proxy for decentralization site (Model 2, S1 Table) to account for possible differences in management and/or mortality across different sites, and found that HR estimates for age, stage and TB changed by less than 10%, and remained statistically significant. We examined the subgroup of patients who did not have CD4 cell counts tested (Model 3, S1 Table) and found that HR estimates for age, stage, and TB changed little relative to the overall cohort, and remained statistically significant. We included two-way interaction terms (Model 4, S1 Table) and found that these were not statistically significant (p>0.05 for all coefficients for two-way interaction terms). Therefore, we chose not to include interaction terms in the primary model for simplicity and model parsimony. We examined the subgroup of children under 5 years of age, and included wasting as an important predictor of survival in the univariate analysis for younger children (Model 5, S1 Table). Wasting remained a significant predictor in the multivariate model (HR 2.1, 95%CI 1.1–4.1, p = 0.020) along with age and stage, whereas TB was no longer significant in this model.

**Fig 2:**
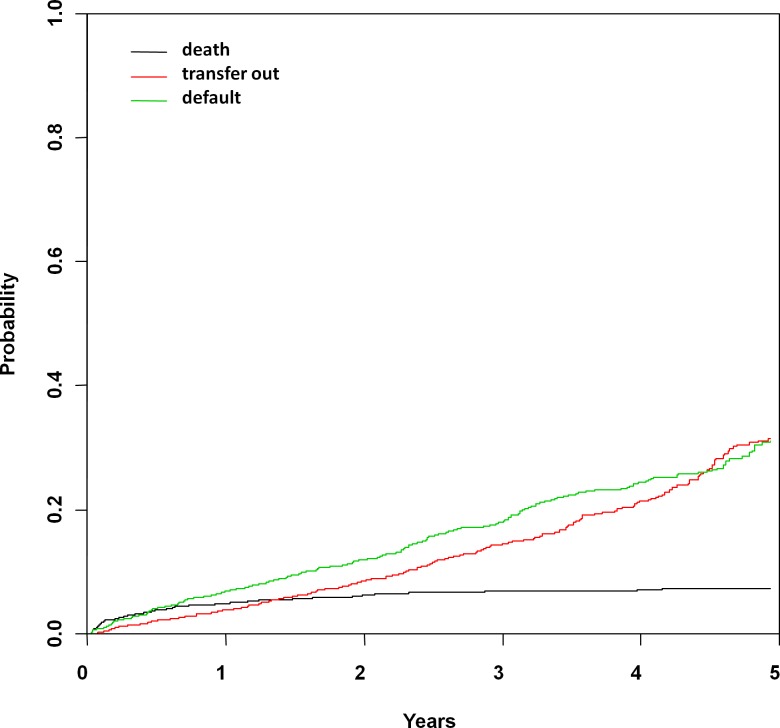
Estimated cumulative incidence curves with mortality, transfer out of cohort, and default as competing events.

Competing risk modeling of the risks of death, transfer out of cohort, and defaulting was performed, and estimated cumulative incidence of these competing events are shown in Fig 2. Table 3 shows the estimated cumulative incidence of death, transfer out of cohort, and defaulting at 1, 3 and 5 years in the competing events model. The cumulative incidence of mortality in the cohort after 1, 3, and 5 years was estimated at 5.2%, 7.1%, and 7.7%, respectively. In competing risk models, mortality differed significantly across age strata (p<0.0001), TB (p = 0.004), and HIV stage (p<0.0001).

**Table 3:** Estimated point-wise cumulative incidence (95% confidence interval) of death, transfer out of cohort, and defaulting, based on competing risk models.

	Year 1	Year 3	Year 5
**Death (entire cohort)**	5.2 (4.2–6.2)	7.1 (6.9–8.4)	7.7 (6.4–9.1)
Age at ART initiation^1^			
<1 year	11 (7.0–16)	14 (9.4–20)	14 (9.4–20)
1-<2 years	6.9 (4.3–10)	9.6 (6.3–14)	12 (7.0–17)
2-<5 years	4.5 (3.0–6.5)	6.9 (4.8–9.5)	6.9 (4.8–9.5)
5–15 years	3.9 (2.8–5.2)	5.1 (3.8–6.7)	5.7 (4.2–7.6)
WHO stage at ART initiation^2^			
I	3.4 (1.8–5.8)	3.8 (2.0–6.4)	3.8 (2.1–6.4)
II	1.8 (0.59–4.2)	2.6 (0.93–5.8)	2.6 (0.93–5.8)
III	4.9 (3.6–6.6)	7.3 (5.5–9.4)	8.0 (6.0–10)
IV	10 (7.2–14)	14 (9.7–18)	15 (10–20)
Tuberculosis at ART initiation^3^			
No TB	4.7 (3.7–5.8)	6.3 (5.1–7.7)	6.6 (5.3–8.1)
TB	7.1 (4.9–10)	10 (7.4–14)	11 (8.2–15)
**Transfer out of cohort (entire cohort)**	4.1 (3.2–5.0)	15 (13–17)	33 (29–37)
**Default (entire cohort)**	7.1 (6.0–8.4)	18 (16–20)	31 (28–35)

^1^ P<0.0001 for difference across age strata.

^2^ P<0.0001 for difference between HIV stages.

^3^ P = 0.004 for difference between TB and no TB.

## Discussion

This study presents outcomes from a large population-based cohort of children receiving care in resource-limited southern Malawi over an 8-year period. The use of routinely collected surveillance data from a national ART program should be considered a strength with respect to providing highly relevant results that can inform pediatric HIV policy in countries highly endemic for HIV infection. In our cohort, the cumulative probability of death was estimated to be 5.3% at 1 year after enrolment; similarly, the 12-month mortality rate was 6.6 deaths per 100 PYs of follow-up. These results are similar to other large pediatric cohort studies in SubSaharan Africa [19–24], but higher than rates found in mature Asian pediatric ART cohorts [25, 26]. A review by Peacock-Villada et al [27] synthesizing and comparing clinical outcomes from published studies of pediatric ART programs in resource-limited versus developed countries found a similar overall mean mortality of 7.6% among 30 cohorts from resource-limited countries, and 7.4% among African cohorts specifically, with most cohorts reporting on approximately 12 months of follow up time.

Our mortality incidence rate of 3.4 deaths per 100 PYs however was somewhat lower than Peacock-Villada’s overall rate of 8.0 and African country rate of 7.5 deaths per 100 PYs. This contrasts with other large cohort reports from east [21] and southern Africa [20] that found similar rates to ours with 3.2 and 2.25 deaths per 100 PYs, respectively. The increased mortality rate estimate in Peacock-Villada’s report may have be influenced by a combination of shorter follow-up times in many studies and known higher mortality in the first year of ART [28]. Our overall mortality incidence rate reflected this reality, with significant differences in mortality across age groups and higher hazard of death amongst infants <1 year old (HR 2.7) and 1 to <2 years old (HR 1.8) compared with older children 5–15 years old. Infants < 1 year likewise had a shorter time to death than the older age groups (60 vs 108 days, respectively). These results highlight the importance of early diagnosis and treatment for infants < 2 years.

The combination of these findings with the trend towards higher mortality we noted in children 2 to <5 years old provides soft support for the 2013 WHO recommendations for universal treatment of children <5 years. While firm evidence suggesting better health outcomes with empiric treatment in this older group compared with immunologic or clinical indications is currently lacking, operational advantages may favour this approach. Only 10% (250/2203) of our cohort had baseline CD4 testing prior to initiation of ART, reflecting the reality of attempting to provide ART in resource-limited settings and the need for pediatric assessment skill in determining clinical disease staging for children. While availability of flow cytometers for CD4 count and percentage measurement has improved steadily, particularly with the roll-out of point-of-care technology in recent years [29], the lag in pediatric ART uptake remains. By broadening ART initiation criteria, the operational barriers of specialized technology and skill can be resolved and hopefully result in better coverage of HIV-infected children in need of ART.

Tuberculosis diagnosis at baseline was associated with lower survival in our cohort, similar to a previous study in Uganda [30], but different from a Malawian study [31] which found no difference in survival in children with or without tuberculosis at the time of ART initiation or previously. There are multiple plausible explanations for the role of tuberculosis in patient deaths, including more advanced forms of tuberculosis due to immune deficiency, drug-drug interactions impacting ART effectiveness, or adverse outcomes or discontinuations due to the burden of medications or medical follow-up. Being signficantly malnourished or wasted at baseline was also associated with lower survival. This is a well-known risk factor for mortality among HIV-infected children [19, 23, 24, 32]. Treatment of severe malnutrition combined with prompt ART initiation has been shown to improve outcomes in affected patients [29], and thus co-delivery of nutrition and HIV services is an essential component of pediatric care programs. Of note, in our supplementary multivariable model that included wasting and TB, wasting but not TB was a significant independent predictor of mortality, suggesting that under-nutrition may be an important mediator of TB-related mortality.

It was interesting to note that there was no impact of year of entry into the cohort, as seen in other studies [19], as one might have expected improved outcomes over time related to increased expertise and quality of care with program maturation. This lack of time effect may reflect changes in patient characteristics due to other programmatic impacts, such as the fact that PMTCT may have decreased the number of new infected infants and improved the outcomes for infants diagnosed earlier through EID programs, thus increasing the proportion of new patients entering at an older age and more advanced stage of illness.

Rates of patients defaulting, or being “lost to follow up” (LTFU) are increasingly recognized as important outcomes to understand in HIV cohort surveillance. Amongst patients categorized as defaultted or LTFU, a number of actual outcomes may be contributing, including death, ART discontinuation, registration at another ART site, or alternate acquisition of ART (eg. private purchase). We found that 7.1%, 18% and 31% of children in our cohort defaulted at 1, 3 and 5 years, respectively. These rates are higher than those seen in most other paediatric cohort studies from Africa) [20–22, 24, 27, 33]. One exception was McNairy et al [19], who found somewhat similar rates of LFTU at 12 (16%) and 24 months (22%) after ART initiation in a review of several sub-Saharan countries. This and other pediatric cohort studies [33] have found LTFU among children to be associated with the same risk factors as for mortality–age < 1 year, recent ART initiation, underweight for age, and advanced HIV disease. When actual patient outcomes are ascertained amongst those LTFU through tracing investigations, death is in fact a common outcome. A systematic review and meta-analysis of studies of mortality amongst adult patients LTFU found an overall combined mortality of 40% [34]. Two Malawian studies [35, 36] similarly found that 41–50% of adult patients and 33% of children who were traced had died. It is likely also to be the case in our study that mortality plays a role among those LTFU; we note that the predictors of death in the remainder of our cohort (younger age and advanced HIV stage) were also predictive of defaulting. It may be that there are other explanations such as families moving to other districts, or seeking out other treatment programs for personal reasons. That said, the high rate of LTFU in this cohort suggests at the very least that the actual mortality rate is higher than what we are able to report with certainty.

This study has several limitations. First is the issue of incomplete data, such as only 10% of patients having CD4 measured at baseline and no documentation of CD4%. However, clinical outcome data was available for >99% of patients, which was the primary outcome of interest. Despite scale up of CD4 and viral load measurement capacity in HIV endemic countries, many programs will none the less still have these same limitations, and relying on clinical assessment parameters will remain a reality in such limited resource settings. We believe our results will reflect those realities and provide insights for such programs. The high rate of loss to follow up also leaves us with uncertainty regarding the true mortality rate in the cohort. As discussed above, this is a common problem that needs to be systematically addressed in scale up of pediatric ART programs in order to gain better understanding of the true outcomes of such programs.

Another limitation is that despite the opportunity to comment on the impact of decentralization of pediatric care on HIV outcomes, we were unable to obtain details of decentralization with respect to pediatric HIV outcomes in our cohort due to limitations within our programmatic surveillance data. Decentralization may have impacted health outcomes, though it is not clear if this may have been positive or negative. Our group’s previous examination of outcomes of decentralized patients [12] showed lower rates of mortality and LTFU in this group, but pediatric patients were a minority (10%) and unevenly distributed with a lower percentage of children in the decentralized group. With recent enhancements to our surveillance system, we are hopeful that in the future we will be able to parse out the impact of decentralization on the pediatric population and add more evidence [37] of the safety and advantages of such care delivery approaches.

Finally, we verified the robustness of conclusions from our statistical models in numerous ways (S1 Table, multivariable models, subgroup analyses, competing risk models), and findings were robust to multiple statistical approaches in that age, HIV stage, and TB remained significant predictors of mortality. Although Kaplan-Meier analyses and Cox proportional hazard models of mortality may be subject to potential biases due to non-informative right censoring due to transfer out of the cohort and defaulting, we also found similar results in competing risk models, which accounted for dropout from the cohort due to transfer and defaulting.Other model assumptions (proportional hazard) were verified using formal statistical tests.

In conclusion, our analysis of this community-based cohort found 1, 3, and 5-year cumulative incidence of mortality of 5.2%, 7.1%, and 7.7%, respectively, with younger age at ART initiation, more advanced clinical stage of HIV, and baseline diagnosis of TB all being associated with mortality. Deaths were disproportionate amongst infants <1 year of age, with 19% of all the deaths occurring in this age group even though they represented only 10% of the cohort. Although prioritized by policy makers, children in ART programs in resource-limited settings still lag behind in accessing HIV care; our results underscore the vulnerability of this group and the need for continued strengthening of efforts to provide them the care they need.

## Supporting Information

S1 Table Sensitivity analysis on multivariable Cox proportional hazard model for survival in the Z-OCS pediatric cohort. (DOCX)
