# Supplementary material for: Survival Outcomes in a Pediatric Antiretroviral Treatment Cohort in Southern Malawi
Source: PLoS One. 2016 Nov 3;11(11):e0165772. doi: 10.1371/journal.pone.0165772 (PMC5094712; doi:10.1371/journal.pone.0165772)
Supplement: S1 Table — (DOCX) [file pone.0165772.s001.docx]

**Table S1. Sensitivity analysis on multivariable Cox proportional hazard model for survival.**

| **Baseline characteristics** | **Unadjusted**  **HR (95% CI)** | **p-value** | **Model 1^1^**  **HR (95% CI)** | **p-value** | **Model 2^2^**  **HR (95% CI)** | **p-value** | **Model 3^3^**  **HR (95% CI)** | **p-value** | **Model 4^4^**  **HR (95% CI)** | **p-value** | **Model 5^5^**  **HR (95% CI)** | **p-value** |
| --- | --- | --- | --- | --- | --- | --- | --- | --- | --- | --- | --- | --- |
| Gender |  |  |  |  |  |  |  |  |  |  |  |  |
| - Female | 1.0 (Reference) |  |  |  |  |  |  |  |  |  |  |  |
| - Male | 1.1 (0.81-1.6) | 0.46 |  |  |  |  |  |  |  |  |  |  |
| Age at ART initiation |  |  |  |  |  |  |  |  | 0.75 (0.59-0.95)^4^ | 0.016 |  |  |
| - <1 year | 3.0 (1.9-4.9) | <0.001 | 2.7 (1.6-4.8) | <0.001 | 2.8 (1.6-4.9) | <0.001 | 2.6 (1.5-4.6) | 0.001 |  |  | 2.1 (1.1-4.0) | 0.020 |
| - 1-<2 years | 1.9 (1.2-3.1) | 0.006 | 1.8 (1.1-3.1) | 0.023 | 1.8 (1.1-3.1) | 0.026 | 1.9 (1.1-3.2) | 0.021 |  |  | 1.5 (0.83-2.7) | 0.19 |
| - 2-<5 years | 1.2 (0.80-1.9) | 0.34 | 1.4 (0.86-2.2) | 0.18 | 1.4 (0.86-2.1) | 0.18 | 1.4 (0.85-2.2) | 0.19 |  |  | 1.0 (Reference)^5^ |  |
| - 5-15 years | 1.0 (Reference) |  | 1.0 (Reference) |  | 1.0 (Reference) |  | 1.0 (Reference) |  |  |  |  |  |
| Calendar year at ART initiation | 0.93 (0.83-1.04)^4^ | 0.18 |  |  |  |  |  |  |  |  |  |  |
| - 2003-5 |  |  |  |  |  |  |  |  |  |  |  |  |
| - 2006 |  |  |  |  |  |  |  |  |  |  |  |  |
| - 2007 |  |  |  |  |  |  |  |  |  |  |  |  |
| - 2008 |  |  |  |  |  |  |  |  |  |  |  |  |
| - 2009 |  |  |  |  |  |  |  |  |  |  |  |  |
| - 2010 |  |  |  |  |  |  |  |  |  |  |  |  |
| - 2011 |  |  |  |  |  |  |  |  |  |  |  |  |
| Site |  |  |  |  |  |  |  |  |  |  |  |  |
| - Chikowi | 1.0 (Reference) |  |  |  | 1.0 (Reference) |  |  |  |  |  |  |  |
| - Mwambo | 1.1 (0.72-1.8) | 0.57 |  |  | 1.2 (0.74-2.1) | 0.43 |  |  |  |  |  |  |
| - Mlumbe | 0.96 (0.58-1.6) | 0.86 |  |  | 1.1 (0.62-1.9) | 0.78 |  |  |  |  |  |  |
| - Malemia | 0.83 (0.44-1.6) | 0.56 |  |  | 0.99 (0.52-1.9) | 0.98 |  |  |  |  |  |  |
| - Kumtumanji | 1.3 (0.70-2.3) | 0.45 |  |  | 1.3 (0.72-2.5) | 0.36 |  |  |  |  |  |  |
| - Zomba Town | 1.8 (0.56-5.8) | 0.32 |  |  | 2.1 (0.64-6.8) | 0.23 |  |  |  |  |  |  |
| - Other^4^ | 0.63 (0.19-2.0) | 0.43 |  |  | 0.46 (0.11-1.9) | 0.28 |  |  |  |  |  |  |
| - unknown | 0.59 (0.14-2.4) | 0.47 |  |  | 0.56 (0.076-4.1) | 0.57 |  |  |  |  |  |  |
| WHO stage at ART initiation |  |  |  |  |  |  |  |  | 1.3 (0.96-1.8)^4^ | 0.085 |  |  |
| - I | 1.0 (Reference) |  | 1.0 (Reference) |  | 1.0 (Reference) |  | 1.0 (Reference) |  |  |  | 1.0 (Reference) |  |
| - II | 0.61 (0.21-1.7) | 0.35 | 0.78 (0.27-2.2) | 0.64 | 0.77 (0.27-2.2) | 0.63 | 0.95 (0.32-2.8) | 0.93 |  |  | 0.86 (0.23-3.1) | 0.82 |
| - III | 1.8 (0.95-3.3) | 0.073 | 1.5 (0.79-3.0) | 0.21 | 1.5 (0.77-2.9) | 0.24 | 1.8 (0.87-3.6) | 0.12 |  |  | 1.8 (0.84-3.8) | 0.14 |
| - IV | 3.9 (2.1-7.4) | <0.001 | 4.0 (2.1-7.7) | <0.001 | 4.0 (2.1-7.6) | <0.001 | 4.9 (2.4-9.8) | <0.001 |  |  | 2.4 (1.1-5.3) | 0.035 |
| - Missing |  |  |  |  |  |  |  |  |  |  |  |  |
| Nutrition status at ART initiation |  |  |  |  |  |  |  |  |  |  |  |  |
| - Wasting^1^ | 3.2 (1.7-5.9)^4^ | <0.001 |  |  |  |  |  |  |  |  | 2.1 (1.1-4.1) | 0.020 |
| - Stunting^2^ | 1.3 (0.74-2.3) | 0.36 |  |  |  |  |  |  |  |  |  |  |
| - Wt-for-age<-3SD | 2.3 (1.4-3.6) | <0.001 |  |  |  |  |  |  |  |  |  |  |
| Co-infections at ART initiation |  |  |  |  |  |  |  |  |  |  |  |  |
| - TB | 1.7 (1.1-2.4) | 0.007 | 2.2 (1.4-3.4) | 0.001 | 2.1 (1.4-3.3) | 0.001 | 2.1 (1.3-3.3) | 0.001 | 0.10 (0.008-1.5) | 0.093 | 1.5 (0.87-2.9) | 0.13 |
| - Oral candidiasis | 0.71 (0.39-1.3) | 0.26 |  |  |  |  |  |  |  |  |  |  |
| Interaction terms |  |  |  |  |  |  |  |  |  |  |  |  |
| - age*stage |  |  |  |  |  |  |  |  | 1.1 (0.99-1.1) | 0.096 |  |  |
| - TB*age |  |  |  |  |  |  |  |  | 1.1 (0.99-1.2) | 0.070 |  |  |
| - TB*stage |  |  |  |  |  |  |  |  | 2.1 (0.94-4.6) | 0.083 |  |  |

^1^

^1^Model 1 is the preferred, final model. Model 1 included age (categorical, 4 factor levels) , WHO stage (categorical, 4 factor levels), and TB (binary) as independent variables in the Cox-proportional hazard model. Participants were censored at the point of last contact for loss to follow-up, transfer out of care, or time of last contact while remaining in the cohort. Selection of variables for inclusion in the model was base on the univariate analysis (included if p<0.1), and backward conditional selection of variables was used to retain variables in the model.

^2^Model 2 included site (categorical, 8 factor levels), age (categorical, 4 factor levels) , WHO stage (categorical, 4 factor levels), and TB (binary) as independent variables in the Cox-proportional hazard model. Participants were censored at the point of last contact for loss to follow-up, transfer out of care, or time of last contact in cohort.

^3^Model 3 is a subgoup analysis of patients who were not tested for CD4 count (n=1981). The patients included in this analysis have a higher mortality, as discussed in the text. Model 3 included age (categorical, 4 factor levels) , WHO stage (categorical, 4 factor levels), and TB (binary) as independent variables in the Cox-proportional hazard model. Participants were censored at the point of last contact for loss to follow-up, transfer out of care, or time of last contact in cohort.

^4^Model 4 includes two-way interaction terms. Because of the large number of two-way interaction terms between dummy variables for categorical variables, we used age and stage as continuous variables in this model. Thus, the hazard ratios should be interpreted as the change in hazard function with a unit change in age (1 year) or stage, assume a linear change in hazard function with change in age or stage, and are not directly comparable in magnitude to Model 1. Model 4 included age in years (continuous) , WHO stage (continuous), and TB (binary), along with 2 way-interaction terms between variables as independent variables in the Cox-proportional hazard model. Participants were censored at the point of last contact for loss to follow-up, transfer out of care, or time of last contact in cohort. None of the coefficients for the 2-way interactions between variables (categorical with multiple factor levels) were statistically significant (p>0.05 for all coefficients).

^5^Model 5 is a subgoup analyis of patients under 5 years of age (n=1146). Model 5 included age (categorical, 3 factor levels) , WHO stage (categorical, 4 factor levels), wasting (binary), and TB (binary) as independent variables in the Cox-proportional hazard model. Participants were censored at the point of last contact for loss to follow-up, transfer out of care, or time of last contact in cohort. Because the reference age group in Model 1 (5-15 years) was not included in Model 5, a different reference age group was used. Thus, the HRs for age categories are not directly comparable to Model 1.
